# A Hydrophilic Polyethylene Glycol-Blended Anion Exchange Membrane to Facilitate the Migration of Hydroxide Ions

**DOI:** 10.3390/polym16111464

**Published:** 2024-05-22

**Authors:** Huaiming Gao, Chenglou Jin, Xia Li, Yat-Ming So, Yu Pan

**Affiliations:** 1 Institute of Functional Textiles and Advanced Materials, College of Textiles and Clothing, State Key Laboratory of Bio-Fibers and Eco-Textiles, Qingdao University, Qingdao 266071, China; 2 Department of Chemistry, The Hong Kong University of Science and Technology, Clear Water Bay, Kowloon, Hong Kong, China

**Keywords:** anion exchange membrane, blended membrane, PEG, ion-conducting

## Abstract

As one of the most important sources for green hydrogen, anion exchange membrane water electrolyzers (AEMWEs) have been developing rapidly in recent decades. Among these components, anion exchange membranes (AEMs) with high ionic conductivity and good stability play an important role in the performance of AEMWEs. In this study, we have developed a simple blending method to fabricate the blended membrane ImPSF-PEGx via the introduction of a hydrophilic PEG into the PSF-based ionic polymer. Given their hydrophilicity and coordination properties, the introduced PEGs are beneficial in assembling the ionic groups to form the ion-conducting channels. Moreover, an asymmetric structure is observed in ImPSF-PEGx membranes with a layer of finger-like cracks at the upper surface because PEGs can act as pore-forming agents. During the study, the ImPSF-PEGx membranes exhibited higher water uptake and ionic conductivity with lower swelling ratios and much better mechanical properties in comparison to the pristine ImPSF membrane. The ImPSF-PEG1000 membrane showed the best overall performance among the membranes with higher ionic conductivity (82.6 mS cm^−1^ at 80 °C), which was approximately two times higher than the conductivity of ImPSF, and demonstrated better mechanical and alkaline stability. The alkaline water electrolyzer assembled by ImPSF-PEG1000 achieved a current density of 606 mA cm^−2^ at 80 °C under conditions of 1 M KOH and 2.06 V, and maintained an essentially unchanged performance after 48 h running.

## 1. Introduction

Hydrogen energy, as an important environmentally friendly and clean energy source, is considered to possess high energy density and emit no carbon emissions, and has become a promising alternative to fossil fuels [1,2,3,4]. Unlike conventional methods reliant on fossil fuels with carbon emissions, green hydrogen is generated through processes that generate zero or minimal carbon emissions, such as water electrolysis technology [5,6]. Compared to proton exchange membrane water electrolyzers (PEMEWs), the operating environment of anion exchange membrane water electrolyzers (AEMWEs) is alkaline and with higher redox reaction kinetics [7,8]. Moreover, their relatively mature stack assembly processes and utilization of non-precious metal catalysts significantly lower their manufacturing costs, which is crucial for their potential commercial application [9,10].

As one of the key components, anion exchange membranes (AEMs) play a vital role in the performance of AEMWEs [11,12]. AEMs with high conductivity and durability are a requirement for high-performing AEMWE equipment. Generally speaking, the ionic conductivity of AEMs can efficiently increase with increasing ionic exchange capacity (IEC). However, the mechanical and alkaline stability of AEMs would reduce after excessive water absorption due to the increase of IEC. Therefore, some strategies have been developed to improve the performance of AEMs by developing the basic polymer materials [13,14], the topology of basic polymers [15,16], ion-exchange groups [17,18], and so on [19,20,21]. For example, He et al. developed a series of highly conductive anion exchange membranes with branched ionic clusters for fuel cells [22]. The improved cation density and cation mobility derived from the branched ionic clusters resulted in highly ordered nano-scale channels for hydroxide ion transport. Hu et al. reported a series of AEMs based on poly(arylene ether nitrile)s with high-density quaternary ammonium cationic side chains [23]. The hydroxide conductivity of the obtained AEMs was recorded in the range of 76.13–119.72 mS cm^−1^ at 80 °C. The assembled water electrolyzer showed that the current density was 521.3 mA cm^2^ at 2.0 V and remained at 500 mA cm^−2^ in a 0.1 M KOH for 480 h. The polybenzimidazole-based AEMs grafted with amphiphilic side chains were produced and showed comb-shaped amphiphilic microphase networks, which achieved a high conductivity of about 91.2 mS cm^−1^, an extremely low swelling ratio of about 8.1% at 80 °C, and good mechanical properties in a hydrated state [24]. A series of multiblock AEMs based on ether-free poly(biphenyl ammonium-b-biphenyl phenyl)s were developed, which could achieve outstanding conductivity of hydroxide (162.2 mS cm^−1^ at 80 °C) with a low swelling ratio, good alkaline stability, and excellent mechanical durability [25].

Apart from the strategies for developing the structures of basic polymers and ion-exchange groups, the ion transport channels can also be efficiently constructed by introducing interactions among basic polymers or functional groups. A new type of AEM with crown ether macrocycles in its main chain was prepared via a one-pot superacid catalyzed reaction [26]. Owing to the self-assembly of crown ethers, this AEM exhibited an obvious phase-separated structure, and its conductivity of hydroxide was up to 122.5 mS cm^−1^ at 80 °C. The comb-shaped poly(terphenyl piperidone)-based AEMs were prepared by introducing macrocyclic crown ethers into their main chains [27]. The macrocycle dynamically trapped ammonium ions as an anchor, which accelerated the aggregation of ionic phases. Zhao et al. reported a series of poly(acenaphthylenyl aryl piperidinium) membranes, in which the π-π self-assembly of acenaphthylene monomer promoted the aggregation of piperidinium cation and the formation of an efficient ion transport channel [28]. Furthermore, the AEMs containing a macrocyclic host–guest structure with dynamic self-protection were fabricated in this paper, and they exhibited high ionic conductivity (114 mS cm^−1^ at 80 °C), low swelling rate (15.3%), and alkali stability [29].

In recent years, we have also been interested in developing new methods for the construction of ion conduction channels to improve the performance of AMEs. The hydrophilic oligo(ethylene glycol) (OEG) groups were introduced into the functional groups of membranes, which exhibited remarkable improvement in hydroxide conductivity and alkaline stability [30]. Efficient formation of continuous ion-conducting channels was facilitated by the dynamic aggregation of water molecules, aided by the hydrophilic side chains. The star-shaped block copolymers were developed as basic polymers for AEMs, which showed a distinct micro-phase separation morphology [31]. The head-to-head cross-linking structure of star-shaped copolymers is beneficial in assembling the functional groups to form continuous ion-conducting channels, facilitating ionic mobility. Moreover, the membranes with benzimidazolium groups showed superior alkaline stability [32]. The PEGs with low molecular weights were used as cross-linkers for iontronic systems to improve their performance [33], and introducing PEG 400 into crown ether-functionalized polysulfone membranes could also improve their performance [34]. In this work, we have developed a strategy for directly blending hydrophilic polymer polyethylene glycol (PEG) into membranes, introducing the hydrophilic functional groups that can self-assemble to form continuous ion-conducting channels. Furthermore, the PEG can also act as a pore-forming agent to generate the micropore structure of the membrane’s surface, which is favorable for water uptake and maintaining dimensional stability. In comparison to the pristine membrane, the blended membranes exhibits an improvement in hydroxide conductivity, as well as dimensional and alkaline stability.

## 2. Materials and Methods

### 2.1. General Procedures and Materials

The polysulfone (PSF) (*M*_w_ of 80,000 Da) and PEG (*M*_n_: 200, 600, 1000, 2000, and 4000 Da) were purchased from Macklin Biochemical Technology. The 1,2-Dimethylimidazole and SnCl_4_ were purchased from Energy Chemical and used as received. The chloromethyl octyl ether (CMOE) was prepared according to the reported procedure [35]. All other chemicals and reagents were procured commercially and utilized without further purification. The CO_2_-free deionized water was employed throughout this paper.

General procedure for preparation of ImPSF-PEGx membrane. Following our reported procedure [30], imidazolium-functionalized polysulfone (ImPSF) was synthesized via the Menshutkin reaction. The ImPSF-PEGx membrane was prepared as follows: The ImPSF was dissolved in N-methylpyrrolidone (NMP), and PEG of various molecular weights (200, 600, 1000, 2000, and 4000 Da) was added in a proportion of 5% (*w*/*w*) for mixing. Then, the mixing solution was filtered and cast on a clean glass plate and heated to 80 °C to remove the solvent. The chloride-containing membrane was subsequently treated with a 1 M KOH aqueous solution at room temperature for 48 h to facilitate the conversion of chloride ions to hydroxide ions. Subsequently, the membrane was soaked and washed in degassed deionized water for 48 h to remove residual KOH, in order to finally obtain the ImPSF-PEGx blended membrane.

### 2.2. Characterization and Measurements

Chemical structure and morphology characterization. The nuclear magnetic resonance (NMR) spectra were collected by a Bruker AVANCE III HD 400 spectrometer (Bruker, Karlsruhe, Germany). Infrared (IR) spectra were recorded by a Nicolet iS50 FT-IR spectrometer (Thermo Fisher Scientific, Waltham, MA, USA) using the attenuated total reflection mode in the wavelength range of 500–4000 cm^−1^. The morphologies of AEMs were measured by a field emission scanning electron microscope (SEM: JSM-7800F, JEOL, Tokyo, Japan), a transmission electron microscope (TEM: JEM-2100Plus, JEOL, Tokyo, Japan), and an atomic force microscope (AFM: Multimode 8, Bruker, Karlsruhe, Germany). The samples suitable for TEM tests were pretreated as follows: 5 µL of a 0.5 wt% ImPSF-PEGx solution of bromide-formed polyelectrolyte was deposited onto a copper grid. The sample was stained via immersion in 1 M KI aqueous solution for 48 h, and then rinsed by deionized water and dried.

Ion exchange capacity (IEC) and hydration number (*λ*). The determination of IEC was conducted using a back titration method: Initially, the dry membrane in its hydroxide form was immersed in a 0.01 M HCl solution for a duration of 24 h. Subsequently, phenolphthalein was employed as the indicator, and the remaining solution was titrated with a standard solution of 0.01 M NaOH. The IEC of the membrane was calculated using the following formula.

(1) $$\mathrm{IEC}=\frac{\left(V_{b}-V_{s}\right)\cdot c_{\mathrm{HCl}}}{m_{\mathrm{dry}}}\times 1000$$

where *V*_b_ represents the volume of NaOH solution used by the blank sample, *V*_s_ is the volume of NaOH solution consumed by the membrane sample, *c*_HCl_ is the concentration of the HCl solution used, and *m*_dry_ is the mass of the dry membrane sample.

The hydration number (*λ*) was calculated using the following formula.

(2) $$\lambda =\frac{\mathrm{WU}}{M_{H2O}}\times \frac{1000}{\mathrm{IEC}}$$

where WU is the water uptake, *M*_H2O_ represents the molecular mass of water (18.015 g mol^−1^), and IEC denotes the ion exchange capacity by titration.

Hydroxide conductivity. The hydroxide conductivity (*σ*) of the membrane (sample size: 1 cm × 4 cm) was measured using the four-electrode AC impedance method in the frequency range of 1 Hz to 100 kHz. Before measurement, the membrane samples were immersed in deionized water for 48 h and subsequently rinsed multiple times with deionized water to eliminate free hydroxide ions. The conductivity was calculated using the following formula.

(3) $$\sigma =\frac{L}{\mathrm{WdR}}$$

where *L* is the distance between the two voltage-sensing electrodes, *W* is the width of the membrane sample (cm), *d* is the thickness of the membrane sample, and *R* is the AC impedance of the membrane sample.

Water uptake (WU) and swelling ratio (SR). The membranes were immersed in degassed deionized water at room temperature for 24 h to ensure complete hydration, then the wetted membranes were wiped with tissue paper, quickly weighed, and measured; the dry weight and length of each membrane was obtained after drying at 60 °C for 24 h, and the water uptake and swelling ratio were determined by the weight and length changes between the hydrated and dried membranes, which were calculated using the following equations:

(4) $$\mathrm{Water}\;\mathrm{uptake}(\%)=\frac{W_{\mathrm{wet}}-W_{\mathrm{dry}}}{W_{\mathrm{dry}}}\times 100$$

(5) $$\mathrm{Swelling}\;\mathrm{ratio}(\%)=\frac{L_{\mathrm{wet}}-L_{\mathrm{dry}}}{L_{\mathrm{dry}}}\times 100$$

where *W*_wet_ and *W*_dry_ are the weights of wet and dry membrane samples, respectively, and *L*_wet_ and *L*_dry_ are the average lengths of wet and dry membrane samples, respectively. *L*_wet_ = (*L*_wet1_ × *L*_wet2_)^1/2^ and *L*_dry_ = (*L*_dry1_ × *L*_dry2_)^1/2^, in which *L*_wet1_ and *L*_wet2_ represent the lengths and widths of wet membrane samples, respectively, and *L*_dry1_ and *L*_dry2_ represent the lengths and widths of dry membrane samples, respectively.

Mechanical and thermal properties. The mechanical properties of hydrated and dry membranes (with a test area of 20 mm × 10 mm) were tested using an Instron 5967 electromechanical universal testing device (Instron, Norwood, MA, USA) with a loading speed of 5 mm min^−1^ at room temperature. The thermal stability of each membrane was tested by a TGA5500 thermogravimetric analyzer (TA, New Castle, DE, USA). The samples were subjected to heating from 30 °C to 700 °C at a rate of 10 °C min⁻¹ in a nitrogen atmosphere to generate a curve. Prior to measurement, the membrane samples underwent drying in a vacuum at 60 °C for 24 h.

Alkaline stability. Each membrane sample was immersed in a solution of 1 M KOH at 80 °C for a designated time, followed by thorough washing and soaking in degassed deionized water for at least 24 h to remove residual KOH. The assessment of the membrane’s alkaline stability involved assessing the alterations in hydroxide conductivity before and after this treatment regimen.

Electrolysis of water. The performance of the ImPSF-PEG1000 membrane in an AEMWE was evaluated. The membrane electrode assembly (MEA) was prepared with a catalyst-coating membrane (CCM). The catalyst, deionized water, isopropyl alcohol, and 75 μL ionomer solution (5 wt% in DMSO) were mixed to prepare a catalyst ink. IrO_2_ and Pt/C were utilized as the anode and cathode catalysts, respectively. The mixture was sonicated in an ice bath for 1 h, and the ink was sprayed on both sides of a membrane to obtain a catalyst-coated membrane. The loading capacities of anode and cathode catalysts were 2.0 mg cm^−2^ and 4 mg cm^−2^, respectively, with nickel foam utilized as the gas diffusion layer. The catalyst-coated membrane, carbon paper in cathode, and titanium paper in anode were then assembled as an MEA. The assembly of the AEMWE cell involved placing the prepared MEA between bipolar plates and securing the plates appropriately. Each bipolar plate is equipped with a single-channel serpentine flow field covering a square area of 2 × 2 cm^2^. A circulation pump supplied the KOH solution to the electrolyzer at a flow rate of 50 mL min^−1^, and polarization curves were recorded using an electrochemical workstation. Polarization curves were generated by sweeping the potential from 1.2 to 2.2 V at a scanning rate of 10 mV s^−1^ at a temperature of 80 °C. The in situ durability of the water electrolysis was evaluated at a constant current density of 400 mA cm^−2^ at 80 °C.

## 3. Results and Discussion

### 3.1. Synthesis and Characterization of ImPSF-PEGx Membrane

The ImPSF was synthesized by a Menshutkin reaction of chloromethylated polysulfone (CMPSF) with 1,2-dimethylimidazole according to a reported procedure [30]. The grafting degree of the synthesized ImPSF was calculated to be 80% according to the integral areas of characteristic peaks in the ^1^H NMR spectra (Figure S1). The blended membranes, ImPSF-PEGx, were fabricated through the mixture of ImPSF and PEGs with different molecular weights, casting, and ion exchanging, in which the “x” is denoted as the number-average molecular weight of PEG. The IR spectra of ImPSF-PEGx membranes showed the characteristic absorption peaks at 2800 cm^−1^, assignable to the C-H stretching peak of the PEG portion (Figure S2).

The fabricated ImPSF-PEGx membranes are transparent and flexible, and the thickness of the membranes can be adjusted by changing the casting solution to about 21 μm (Figure S3). This suggests potential advantages in enhancing the practical operation of the membrane electrode assembly (MEA) [36]. Furthermore, the direct blending of PEG with basic materials provides a convenient approach for the preparation of AEMs with an enhanced performance, which can be scaled up by improving the casting method and equipment. It shows potential for industrial applications thanks to its convenience and relatively low manufacturing costs.

**Figure 1:**
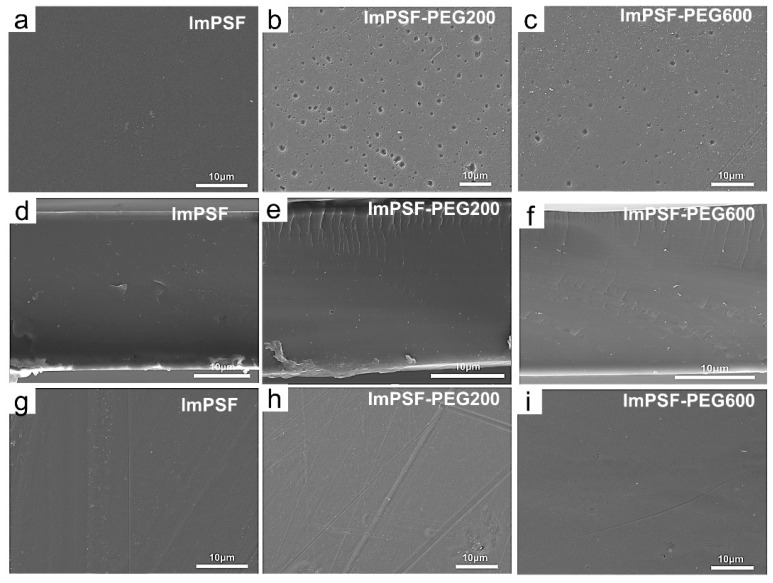
SEM images of ImPSF, ImPSF-PEG200, and ImPSF-PEG600 membranes: (**a**–**c**) upper surfaces; (**d**–**f**) cross-sections; and (**g**–**i**) lower surfaces.

**Figure 2:**
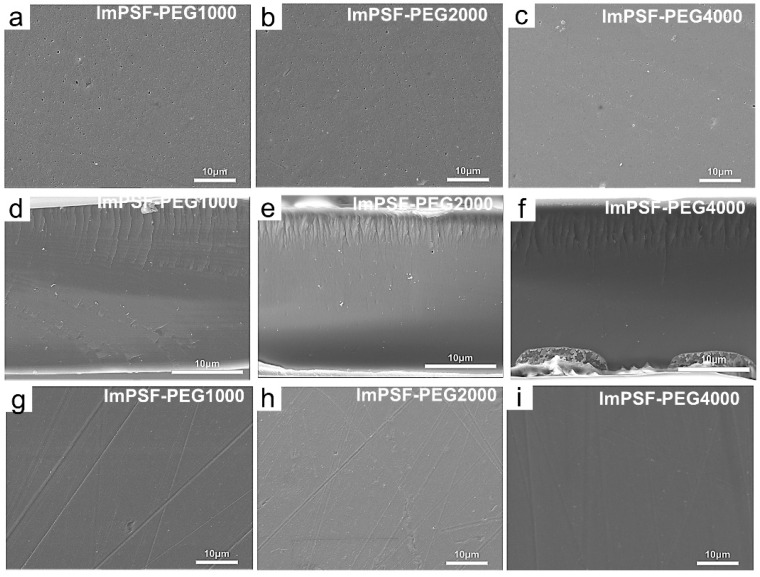
SEM images of ImPSF1000, ImPSF-PEG2000, and ImPSF-PEG4000 membranes: (**a**–**c**) upper surfaces; (**d**–**f**) cross-sections; and (**g**–**i**) lower surfaces.

The surface and cross-sectional morphology of the membrane was characterized with a scanning electron microscope (SEM). As shown in Figure 1 and Figure 2, the ImPSF membrane showed a defect-free surface; in comparison, the ImPSF-PEGx membranes showed a micropore structure at their top surfaces. The average pore sizes on each surface were 0.73 ± 0.22 μm for the ImPSF-PEG200 membrane, 0.65 ± 0.15 μm for the ImPSF-PEG600 membrane, 0.52 ± 0.11 μm for the ImPSF-PEG1000 membrane, and 0.23 ± 0.08 μm for the ImPSF-PEG2000 membrane, but the micropore structure at the surface of ImPSF-PEG4000 was not distinct. The average pore sizes decreased with an increase of the molecular weight of PEG. This might be attributed to the easier extraction of small molecular PEG from the basic polymers that acted as a pore-forming agent [37,38,39]. The asymmetric structures of the ImPSF-PEGx membranes were further characterized by a SEM test of the membrane’s cross-section. As shown in Figure 1d–f and Figure 2d–f, a region of finger-like cracks was displayed at the skin layers of the ImPSF-PEGx membranes. The thickness of the region gradually decreased with the increase of the molecular weight of PEG. The highly porous region of finger-like cracks is beneficial for forming water channels and reducing the resistance of ionic migration. Owing to a low additional amount of PEG (5%), the micropores at the surface of the ImPSF-PEGx membrane were dispersed and non-penetrating. The bottom surface of the ImPSF-PEGx membrane was defect-free and compact (Figure 1g–i and Figure 2g–i).

**Figure 3:**
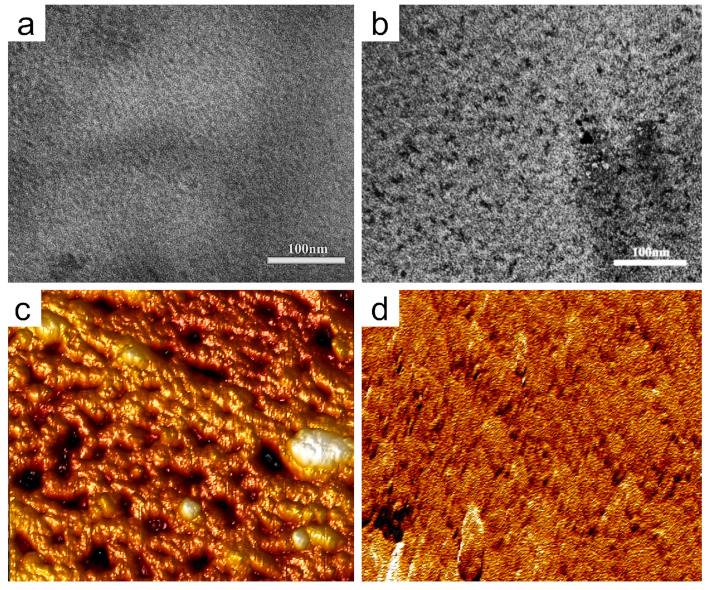
TEM images of ImPSF (**a**) and ImPSF-PEG1000 (**b**), and AFM images of upper surface (**c**) and lower surface (**d**) of ImPSF-PEG1000.

Meanwhile, a transmission electron microscope (TEM) and an atomic force microscope (AFM) were used to study the morphology of the membrane. For the TEM images of the representative membranes, as depicted in Figure 3a,b, the bright domains belong to hydrophobic regions formed by the polymer skeletons, while the dark regions belong to the hydrophilic domains generated by self-aggregation of the cations and water molecules [24,40]. In comparison to ImPSF, distinct hydrophilic clusters were observed in ImPSF-PEG1000. This suggests that the PEG molecules with inherent hydrophilicity promote the aggregation of ionic groups, which is beneficial for constructing effective ion-conducting channels. Furthermore, the AFM analysis of the morphology of ImPSF-PEG1000 revealed the micropore structure at the top surface of the membrane, which was consistent with the observations made during SEM analysis (Figure 3c,d).

### 3.2. Ion Exchange Capacity, Water Uptake, and Swelling Ratio

The ion exchange capacity (IEC), water uptake (WU), and swelling ratio (SR) of each fabricated membrane is shown in Table 1. The IEC is related to the number of functional groups in the ionomers and directly influences WU, SR, and ionic conductivity [41]. The IEC values of the ImPSF and ImPSF-PEGx membranes were almost identical to the theoretical IEC values, indicating the complete conversion of chloromethyl to imidazolium groups.

**Figure 4:**
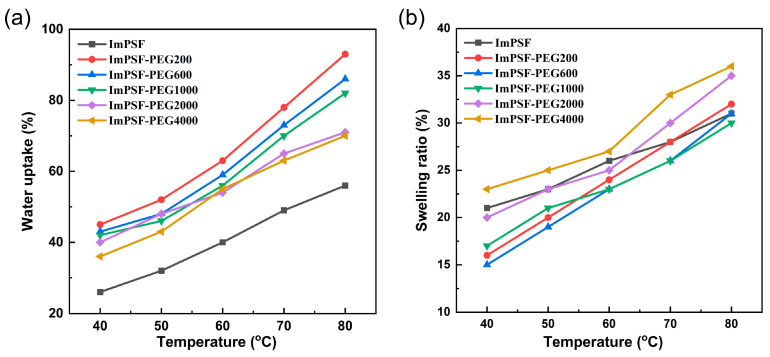
The WU (**a**) and SR (**b**) of ImPSF and ImPSF-PEGx membranes.

The WU values of AEMs significantly impact the ionic conduction, but excess WU can dilute the ionic concentration within the membrane, leading to severe swelling issues [42]. Therefore, it is crucial to design a membrane with good water absorption and a restrained swelling ratio. In comparison to the WU of ImPSF (26%), the ImPSF-PEGx membranes showed higher WU values (36–45%). This was attributed to the blended hydrophilic PEG component and the micropores at the surface, which were beneficial in absorbing and storing water molecules. With an increase in temperature, the WU of the ImPSF-PEGx membranes increased from 36% to 45% at 40 °C, and from 68% to 93% at 80 °C (Figure 4a). The ImPSF-PEG2000 and ImPSF-PEG4000 blended with higher molecular weight PEG displayed relatively lower WU than ImPSF-PEG200 and ImPSF-PEG600, owing to the poor hydrophilic ability of higher molecular weight PEG and fewer micropores at their surfaces [43]. Significantly, the ImPSF-PEGx membranes with higher WU showed a lower or similar SR (16% to 23%) compared to the ImPSF membrane (21%) at 40 °C (Figure 4b), indicating an advantage of blending PEG for the dimensional stability of the membrane. This is probably owing to the finger-like cracks at the surface that can hold water molecules without excessive swelling and maintain dimensional stability [44]. Furthermore, the hydration number (λ) was calculated in order to elucidate the quantity of water molecules carried around the cationic groups. For ImPSF-PEGx membranes at 80 °C, the range of λ was calculated to be 26.2–34.9, which is higher than that of ImPSF membranes.

### 3.3. Hydroxide Conductivity

**Figure 5:**
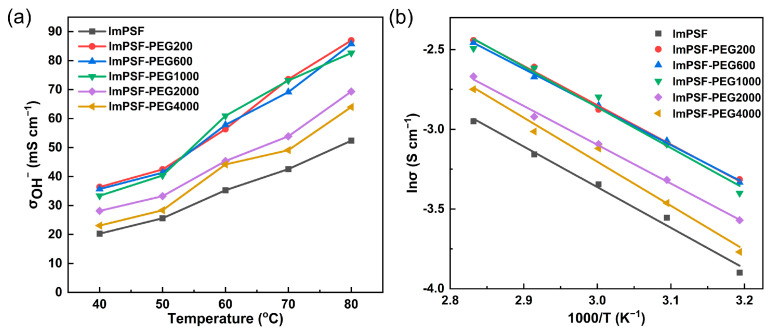
The OH^−^ conductivities as a function of temperature (**a**) and the Arrhenius plots (**b**) of the ImPSF and ImPSF-PEGx membranes: *E*_a_ of 22.39 kJ mol^−1^ for ImPSF; *E*_a_ of 20.46 kJ mol^−1^ for ImPSF-PEG200; *E*_a_ of 20.66 kJ mol^−1^ for ImPSF-PEG600; *E*_a_ of 20.87 kJ mol^−1^ for ImPSF-PEG1000; *E*_a_ of 21.19 kJ mol^−1^ for ImPSF-PEG2000; *E*_a_ of 21.78 kJ mol^−1^ for ImPSF-PEG4000.

Hydroxide conductivity (σ), as a crucial property for evaluating the performance of AEMs, was calculated according to the resistance of AEMs. The hydroxide conductivities of the fabricated membranes at different temperatures are shown in Table 2. The hydroxide conductivity of ImPSF-PEGx membranes is 22.6 to 25.3 mS cm^−1^ at 40 °C, which is higher than that of ImPSF (19.0 mS cm^−1^ at 40 °C). In Figure 5a, owing to increased molecular migration, the hydroxide conductivity of all the fabricated AEMs is shown to increase at elevated temperatures [12]. The ImPSF-PEG200, ImPSF-PEG600, and ImPSF-PEG1000 membranes exhibited relatively higher conductivities than the ImPSF-PEG2000 and ImPSF-PEG4000 membranes. This phenomenon shows that the introduction of PEGs with small to moderate molecular weights could efficiently promote the conductivity of hydroxide. This might be attributed to the region of finger-like cracks at the surface of a membrane reducing the resistance of hydroxide migration. The activation energy (*E*_a_) of hydroxide migration was calculated to be 20.46 to 22.39 kJ mol^−1^ (Figure 5b). The highest *E*_a_ of 22.39 kJ mol^−1^ is for ImPSF, and the lowest *E*_a_ of 2.46 kJ mol^−1^ is for ImPSF-PEG200. This further suggests the importance of blending PEG in reducing the resistance of hydroxide migration [36].

### 3.4. Mechanical and Thermal Stability

**Figure 6:**
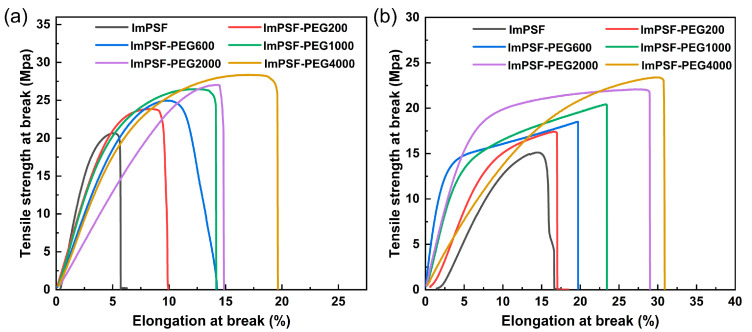
The tensile curves of the ImPSF and ImPSF-PEGx membranes in dry (**a**) and wet (**b**) states.

The mechanical properties of AMEs are closely related to the durability and fabrication of MEA. Therefore, the mechanical properties of ImPSF-PEGx membranes were studied using a universal testing machine in both dry and wet states, and the results are shown in Table 3 and Figure 6. The ImPSF-PEGx membranes showed tensile strength in the range of 23.7 to 28.0 MPa in a dry state and 17.3 to 23.3 MPa in a wet state. Meanwhile, their elongation at breaks is in the range of 9.2 to 19.1% in a dry state and 16.5 to 29.7% in a wet state. In comparison to ImPSF, the ImPSF-PEGx membranes exhibited an improvement of both tensile strength and elongation at breaks, and moreover, the tensile strength and elongation at breaks of the ImPSF-PEGx membranes increased with increases in the molecular weight of PEG [45]. The mechanical properties of AMEs can meet the necessary requirements for electrolyzer applications.

**Figure 7:**
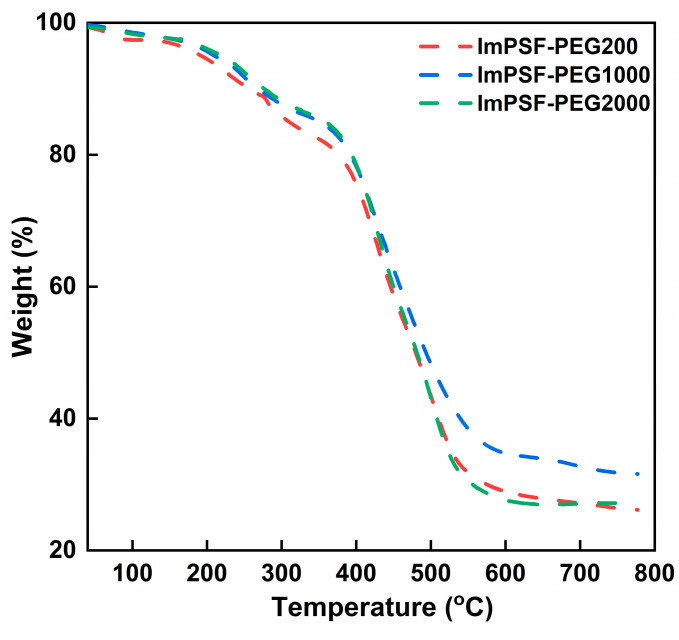
TGA curves of representative ImPSF-PEGx membranes at a temperature increase rate of 10 °C min^−1^ in a nitrogen atmosphere.

The thermal stability of an AEM determines its upper temperature limit while functioning in an anion exchange membrane water electrolyzer (AEMWE) system. The thermal decompositions of representative ImPSF-PEG200, ImPSF-PEG1000, and ImPSF-PEG2000 membranes were tested via thermogravimetric analysis (TGA), with the results shown in Figure 7. Similar thermal degradation curves were observed for the ImPSF-PEGx membranes, which can be roughly divided into three stages. When the temperature increased from 30 to 100 °C, the weight losses of the membranes were less than 5%, which is mainly due to the evaporation of hydration water and residual solvents within the membranes [46]. Then, the onset decomposition temperature (T_OD_) was about 155 °C, and the fastest decomposition temperature (T_FD_1) was about 240 °C. The loss of weight can be attributed to the decomposition of functional groups 1,2-dimethylimidazole and PEG within the membrane. The second-fastest decomposition temperature (T_FD_2) was about 420 °C, and this is due to the decomposition of the polymer backbone [30]. It is shown that the thermal decompositions of ImPSF-PEGx membranes can meet the practical requirements of the electrolyzer [47].

### 3.5. Alkaline Stability

**Figure 8:**
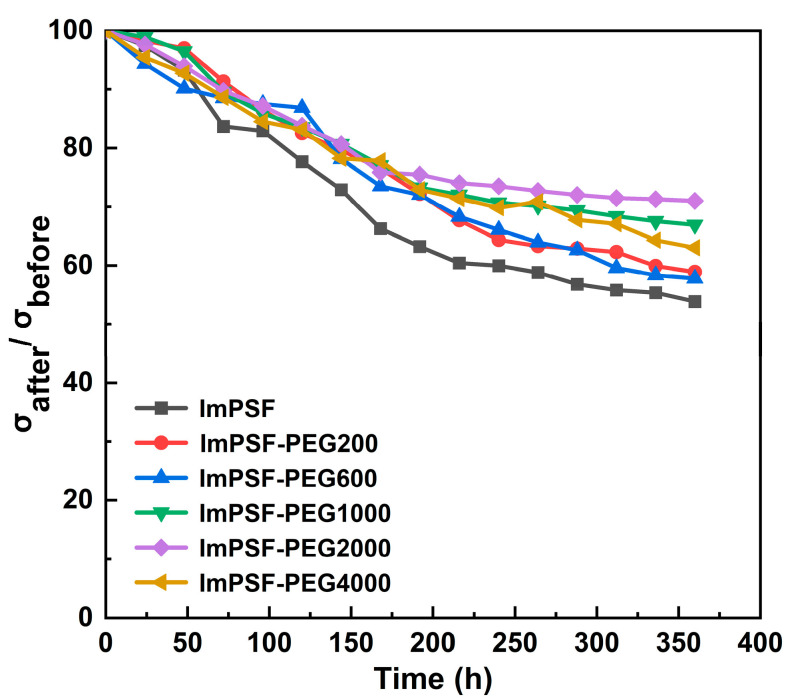
Remaining OH^−^ conductivities of ImPSF and ImPSF-PEGx membranes in 1 M KOH at 80 °C for 360 h.

Owing to the applications involving alkaline conditions, the alkaline stability of AEMs is crucial for the long-term operation of AEMWEs, and therefore, the ImPSF and ImPSF-PEGx membranes were immersed in 1 M KOH solution at 80 °C to investigate their alkaline stability. All membrane samples maintained their integrity and there was no cracking of the membranes during the testing period (Figure S4). As shown in Figure 8, all the prepared AEMs displayed an obvious decrease over time during retained conductivity, suggesting that chemical degradation might occur. This might be attributed to the degradation of imidazolium groups under alkaline conditions at a certain temperature [48,49,50]. It was observed that the degradation initially occurred rapidly, followed by a tendency for the retained conductivity to stabilize. This can be attributed to the direct contact of the KOH solution with the cationic groups on the membrane surface [51]. After that, the degree of solvation of OH^−^ in the membrane matrix increased, which reduced the aggressiveness of OH^−^, so the retained conductivity tended to stabilize [52,53].

It was found that the hydroxide conductivity of ImPSF-PEG200, ImPSF-PEG600, ImPSF-PEG1000, ImPSF-PEG2000, and ImPSF-PEG4000 remained at 59%, 58%, 67%, 71%, and 63% of their original values, respectively, after 360 h (15 days), which showed moderate alkaline stability in comparison to the reported membranes under the same testing conditions [26,54,55,56,57,58]. In comparison, the ImPSF membrane remained at only 52% of its original value after 360 h, showing that blending with PEG can significantly improve the alkaline stability of AEMs. This is probably owing to the interaction between PEG segments and quaternary ammonium groups, which can weaken attacks by hydroxide ions [30]. Furthermore, the ImPSF-PEGx membranes that contain PEG with smaller molecular weights were found to possess relatively poor alkaline stability. This might be caused by the increased water uptake and micropores, which enhance the probability of attacks by hydroxide ions on the polymer backbone and functional groups. To further investigate the degradation behavior of the membrane, the degraded membranes were investigated by NMR spectroscopy, which showed the degradation of imidazolium groups to amide and enamine (Figure S5) [59]. Then, the possible degradation mechanism was proposed (Figure S6).

### 3.6. The Performance of the Alkaline Water Electrolyzer

**Figure 9:**
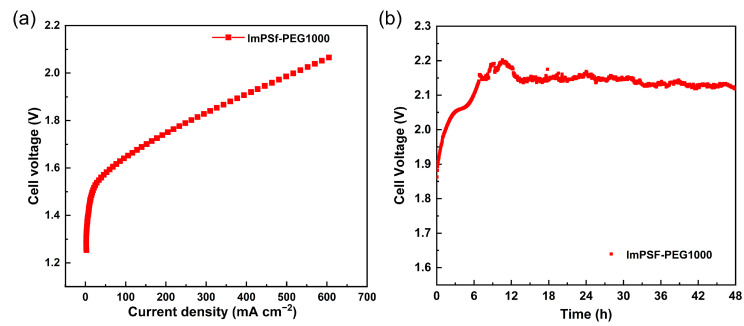
Polarization (**a**) and durability (**b**) curves of AEMWE assembled using ImPSF-PEG1000.

In order to evaluate the effect of ImPSF-PEGx membranes on the performance of AEMWEs, the MEA equipped with the representative ImPSF-PEG1000 membrane was studied, which exhibited good ionic conductivity and stability. As shown in Figure 9, the polarization curve of the AEMWE based on ImPSF-PEG1000 was recorded between 1.2 and 2.2 V. It was found that the current density of 606 mA cm^−2^ was achieved at 2.06 V for the AEMWE with 1M KOH at 80 °C. During continuous operation at a constant current density of 400 mA cm^−2^, the voltage of the electrolytic cell began to increase, then gradually decreased after 10 h, indicating that the activation process had occurred [60]. After 12 h, the voltage stabilized at around 2.1 V with minimal fluctuations. We noted that the performance of the alkaline water electrolyzer equipped with the fabricated membrane was relatively good compared to the reported AEMs (Table S1). It should be mentioned that the performance of the alkaline water electrolyzer varies depending on a range of parameters [61], including catalyst loading, ionomer type, temperature, and flow rate; we will further optimize the preparation method and operating conditions of the electrolysis cell to achieve excellent performance.

## 4. Conclusions

The blended ImPSF-PEGx membranes were fabricated by blending PEGs with PSF-based ionic polymer. The blended PEGs were beneficial in assembling the ionic groups to form ion-conducting channels because of their hydrophilicity and coordination properties. On the other hand, a small amount of PEGs also acted as pore-forming agents to form a layer of finger-like cracks at the upper surface and generate the asymmetric structure of each ImPSF-PEGx membrane. Owing to their asymmetric structures and cracked layers, the blended ImPSF-PEGx membranes, with their higher WU but lower SR, showed better dimensional stability and mechanical properties than the pristine ImPSF membrane. Furthermore, the hydroxide conductivities of ImPSF-PEGx were much higher than those of ImPSF, suggesting the promotion of hydroxide migration by blending PEGs. The ImPSF-PEG200 membrane with the most distinct cracked layer and the highest WU showed the highest ionic conductivity of 87.0 mS cm^−1^ at 80 °C; in comparison, the ImPSF-PEG1000 membrane showed the relative best overall performance among the membranes, with higher ionic conductivity (82.6 mS cm^−1^ at 80 °C) and better alkaline stability. Additionally, the ImPSF-PEG1000 membrane was used to assemble an alkaline water electrolyzer. The electrolyzer exhibited a high current density of 606 mA cm^−2^ at 80 °C under conditions of 1 M KOH and 2.06 V. After operation for 48 h, the performance of the electrolyzer remained essentially unchanged. It is shown that the blending strategy is convenient and efficient in enhancing the performance of AEMs, which provides advantageous insights into reducing ion migration resistance and indicates a potential application in the design of high-performance AEMs.

## Figures and Tables

**Table 1:** IEC, WU, SR, and *λ* of ImPSF and ImPSF-PEGx membranes.

Run	Membrane	IEC ^a^ (mmol g^−1^)		WU ^b^ (%)		SR ^b^ (%)		*λ* ^c^	
		Theo.	Expt.	40 °C	80 °C	40 °C	80 °C	40 °C	80 °C
1	ImPSF	1.56	1.55	26	56	21	31	9.3	19.9
2	ImPSF-PEG200	1.48	1.48	45	93	16	32	16.9	34.9
3	ImPSF-PEG600	1.48	1.46	43	86	15	31	16.4	32.7
4	ImPSF-PEG1000	1.48	1.45	42	82	17	30	16.1	31.4
5	ImPSF-PEG2000	1.48	1.43	40	71	20	35	15.6	27.6
6	ImPSF-PEG4000	1.48	1.44	36	68	23	36	13.9	26.2

^a^ The theoretical IEC values were calculated from the structure and composition of the polymer in OH^−^ form, and the experimental IEC values were measured by the back titration method. ^b^ The WU and SR were assessed based on the changes in weight and length between the hydrated membrane and its dry state at temperatures of 40 °C and 80 °C. ^c^ *λ*: the quantity of adsorbed water molecules per ion-conducting group.

**Table 2:** IEC and hydroxide conductivities of ImPSF and ImPSF-PEGx membranes.

Run	Membrane	IEC (mmol g^−1^)	*σ* (mS cm^−1^)	
			40 °C	80 °C
1	ImPSF	1.56	19.0	43.3
2	ImPSF-PEG200	1.48	25.1	87.0
3	ImPSF-PEG600	1.46	24.6	85.8
4	ImPSF-PEG1000	1.45	25.3	82.6
5	ImPSF-PEG2000	1.43	22.6	61.3
6	ImPSF-PEG4000	1.44	22.7	60.8

**Table 3:** Mechanical properties of ImPSF and ImPSF-PEGx membranes.

Membrane	Tensile Strength at Break (Mpa)		Elongation at Break (%)	
	Dry	Wet	Dry	Wet
ImPSF	20.7	15.1	5.0	14.5
ImPSF-PEG200	23.7	17.3	9.2	16.5
ImPSF-PEG600	24.8	18.5	10.5	19.7
ImPSF-PEG1000	26.1	20.3	13.6	23.4
ImPSF-PEG2000	27.0	21.8	14.5	28.3
ImPSF-PEG4000	28.0	23.3	19.1	29.7

## Data Availability

Data are contained within the article and Supplementary Materials.

## Supplementary Materials

The following supporting information can be downloaded at: https://www.mdpi.com/article/10.3390/polym16111464/s1, Figure S1: ^1^H NMR spectra (500 MHz, 25 °C) of CMPSF (in CDCl_3_) and ImPSF (in *d*-DMSO); Figure S2: FT-IR spectra of PEG1000, ImPSF, and ImPSF-PEG1000; Figure S3: Digital photographs of ImPSF-PEG1000; Figure S4: Digital photographs of ImPSF and ImPSF-PEGx, original (a–e) and after alkaline treatment (f–j); Figure S5: The ^1^H NMR spectra of original ImPSF and alkaline-treated ImPSF; Figure S6: Possible degradation mechanism of imidazolium in KOH solution; Table S1: Comparison of water electrolyzer performance of AEMs. References [26,58,62,63,64,65,66,67,68,69] are cited in Supplementary Material.

## Abbreviations

PEMEWs	exchange membrane water electrolyzers
AEMWEs	anion exchange membrane water electrolyzers
AEMs	anion exchange membranes
PSF	polysulfone
CMPSF	chloromethylated polysulfone
ImPSF	imidazolium-functionalized polysulfone
OEG	oligo(ethylene glycol)
PEG	polyethylene glycol
CMOE	chloromethyl octyl ether
IEC	ionic exchange capacity
WU	water uptake
SR	swelling ratio
MEA	membrane electrode assembly
CCM	catalyst-coating membrane
