# Supplementary material for: A Hydrophilic Polyethylene Glycol-Blended Anion Exchange Membrane to Facilitate the Migration of Hydroxide Ions
Source: Polymers (Basel). 2024 May 22;16(11):1464. doi: 10.3390/polym16111464 (PMC11175046; doi:10.3390/polym16111464)
Supplement: Supplementary file 1 [file polymers-16-01464-s001.zip › polymers-2992927-supplementary.pdf]

Supporting information

A Hydrophilic Polyethylene Glycol-Blended Anion Exchange Membrane to Facilitate the Migration of Hydroxide Ions

Huaiming Gao [1], Chenglou Jin [1], Xia Li [1], Yat-Ming So [2] and Yu Pan [1,*]

[1] Institute of Functional Textiles and Advanced Materials, College of Textiles and Clothing, State Key Laboratory of Bio-Fibers and Eco-Textiles, Qingdao University, Qingdao 266071, China; huaiminggao@163.com (H.G.); jcl990209@163.com (C.J.); lixia0323@foxmail.com (X.L.)
[2] Department of Chemistry, The Hong Kong University of Science and Technology, Clear Water Bay, Kowloon, Hong Kong, China; yatmingso@ust.hk
* Correspondence: ypan@qdu.edu.cn

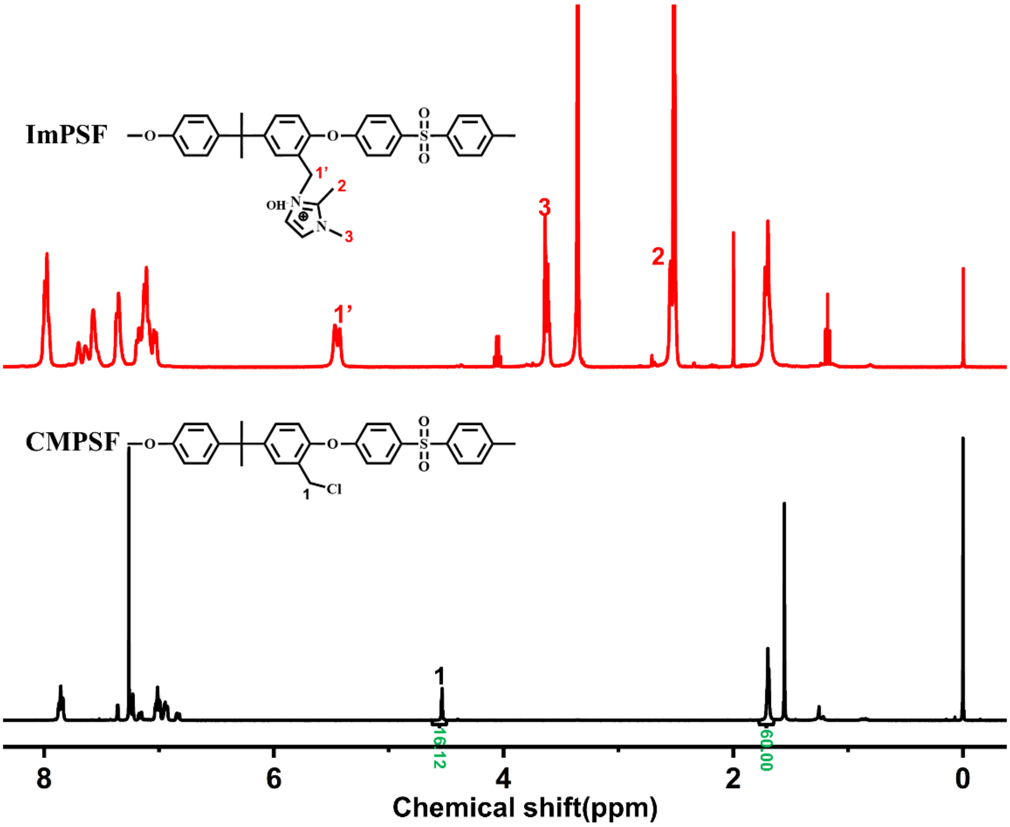

Figure S1. ^1H NMR spectra (500 MHz, 25 °C) of CMPSF (in $CDCl_3$) and ImPSF (in *d*-DMSO).

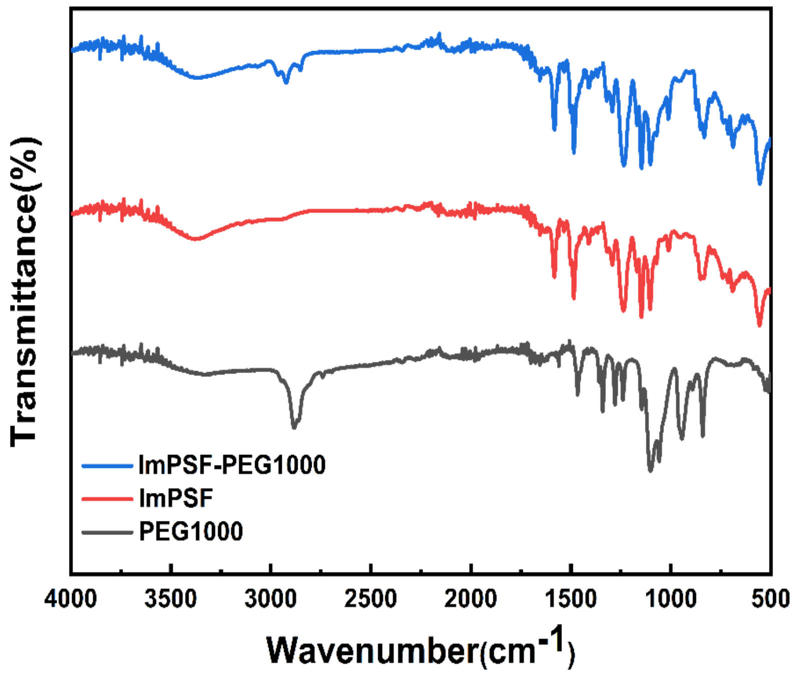

Figure S2. FT-IR spectra of PEG1000, ImPSF and ImPSF-PEG1000.

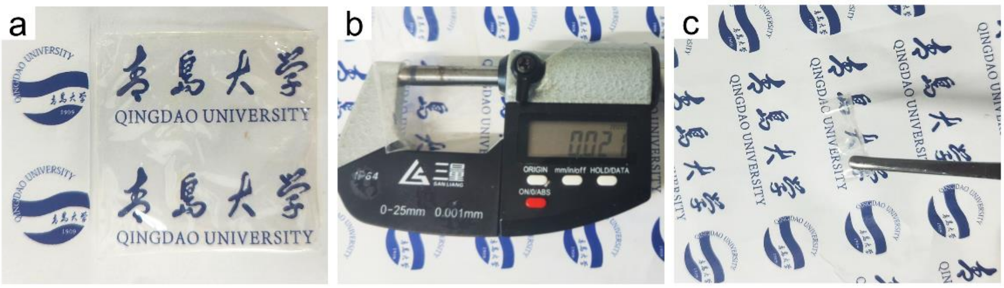

Figure S3. Digital photographs of ImPSF-PEG1000.

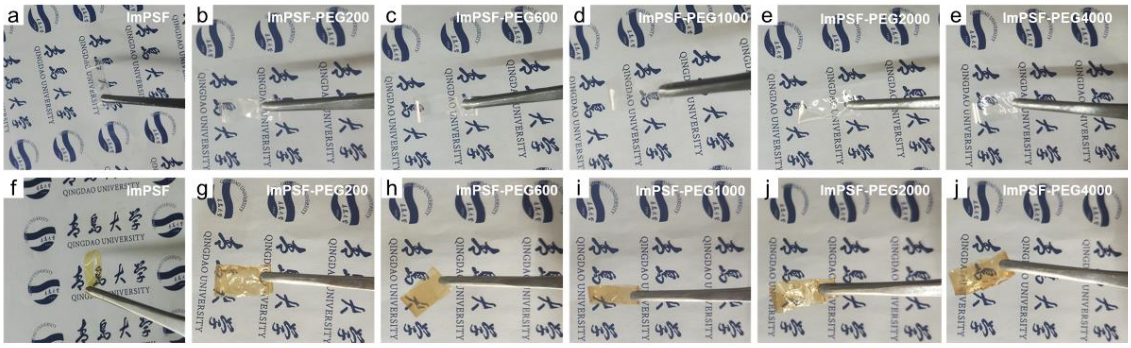

Figure S4. Digital photographs of ImPSF and ImPSF-PEGx original (a–e) and after alkaline treatment (f–j).

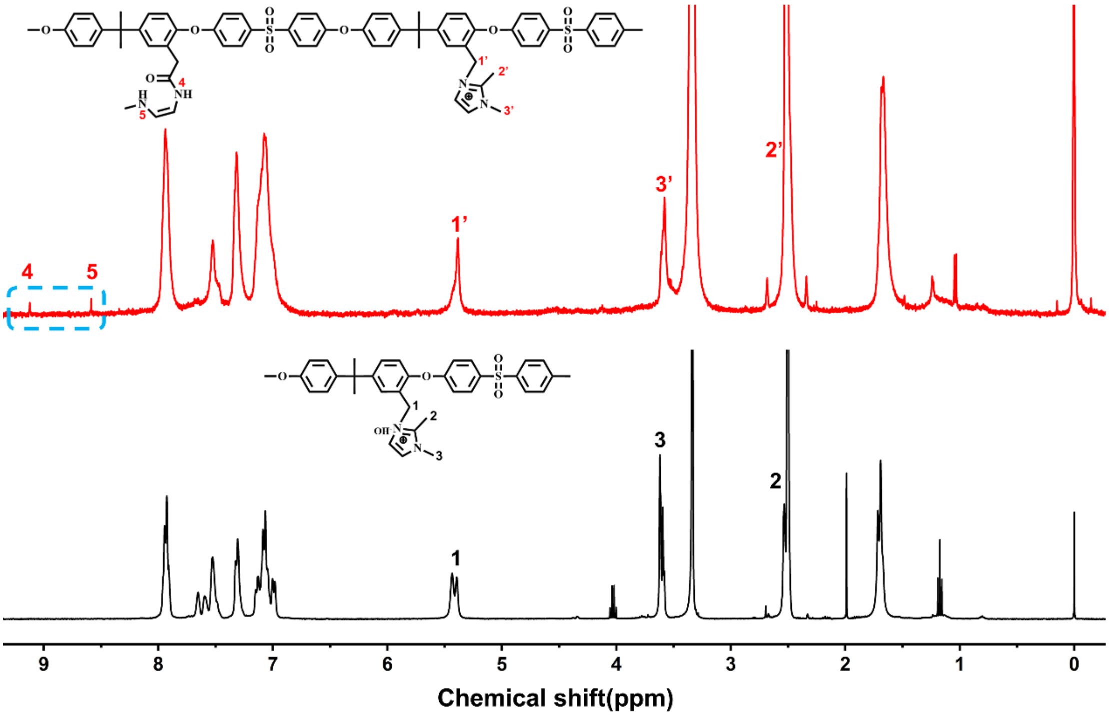

Figure S5. The ^{1}H NMR spectra of original ImPSF and alkaline treated ImPSF.

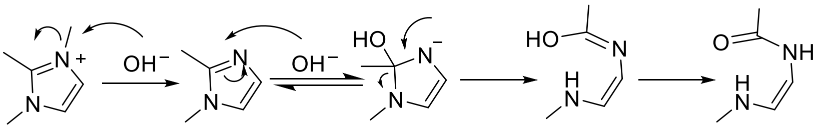

Figure S6. Possible degradation mechanism of imidazolium in KOH solution.

Table S1 Comparison of electrolyzer performance of AEMs.

Membrane	σ (mS cm^{-1})	Catalyst loading		T (°C)	Feed (KOH)	Performance @2 V, mA cm^{-2}	Reference
		Anode	Cathode				
ImPSF-PEG1000	82.6 (80°C)	IrO_2	Pt/C	80	1 M	606	This work
Sustainion® X37-50	90.0 (40°C)	NiFe	Ni	40	1 M	300	[a]
Fumasep FAA-3-50	55.0 (100°C)	$NiMn_2O_4$	Pt/C	80	1 M	530	[b]
PISPVA46	89.7 (60°C)	IrO_2	Pt/C	60	0.5 M	547	[c]
C-IL-100	95.0 (80°C)	NiFeCO	NiFeCO	80	1 M	550	[d]
AF1-HNN8-25	-	NiFe	Ni	60	1 M	620	[e]
PTFE-Sustainions	-	Ni–Fe	Ni–Fe	60	1 M	840	[f]
Co-PAES-QA-25-60	43.0 (80°C)	IrO_2	PtRu/C,	80	2 M	1646	[g]
PDTP-10	110.0 (80°C)	IrO_2	Pt/C	80	1 M	2000	[h]
BNTP-MP11	181.2 (90°C)	IrO_2	Pt/C	80	1 M	2700	[i]
PTP-OEG4	104.2 (80°C)	IrO_2	Pt/C	80	1 M	5900	[j]

References

a. López-Fernández, E.;Gómez-Sacedón, C.;Gil-Rostra, J.;Espinós, J.P.;Brey, J.J.;González-Elipe, A.R.;De Lucas-Consuegra, A.;Yubero, F. Optimization of anion exchange membrane water electrolyzers using ionomer-free electrodes. *Renew. Energy* **2022**, *197*, 1183-1191.

b. Carbone, A.;Zignani, S.C.;Gatto, I.;Trocino, S.;Aricò, A.S. Assessment of the FAA3-50 polymer electrolyte in combination with a NiMn2O4 anode catalyst for anion exchange membrane water electrolysis. *Int. J. Hydrogen Energy* **2020**, *45*, 9285-9292.

c. Park, H.J.;Lee, S.Y.;Lee, T.K.;Kim, H.-J.;Lee, Y.M. N3-butyl imidazolium-based anion exchange membranes blended with Poly(vinyl alcohol) for alkaline water electrolysis. *J. Membr. Sci.* **2020**, *611*, 118355.

d. Wang, X.;Lammertink, R.G.H. Dimensionally stable multication-crosslinked poly(arylene piperidinium) membranes for water electrolysis. *J. Mater. Chem. A* **2022**, *10*, 8401-8412.

e. Qian, J.;Wang, C.;Zhang, X.;Hu, J.;Zhao, X.;Li, J.;Ren, Q. Quaternary ammonium-functionalized crosslinked poly(aryl ether sulfone)s anion exchange membranes with enhanced alkaline stability for water electrolysis. *J. Membr. Sci.* **2023**, *685*, 121946.

f. Khataee, A.;Shirole, A.;Jannasch, P.;Krüger, A.;Cornell, A. Anion exchange membrane water electrolysis using Aemion™ membranes and nickel electrodes. *J. Mater. Chem. A* **2022**, *10*, 16061-16070.

g. Motealleh, B.;Liu, Z.;Masel, R.I.;Sculley, J.P.;Richard Ni, Z.;Meroueh, L. Next-generation anion exchange membrane water electrolyzers operating for commercially relevant lifetimes. *Int. J. Hydrogen Energy* **2021**, *46*, 3379-3386.

h. Wang, X.;Qiao, X.;Liu, S.;Liu, L.;Li, N. Poly(terphenyl piperidinium) containing hydrophilic crown ether units in main chains as anion exchange membranes for alkaline fuel cells and water electrolysers. *J. Membr. Sci.* **2022**, *653*, 120558.

i. Gao, W.;Gao, X.;Choo, Y.;Jun W, J.; Cai, Hong.;Z.;Gen Q.;Mei Z,A.;Liu, Q. Durable dual-methylpiperidinium crosslinked poly(binaphthyl-co-terphenyl piperidinium) anion exchange membranes with high ion transport and electrochemical performance. *Chem. Eng. J.* **2023**, *466*, 143107.

j. Liu, L.;Bai, L.;Liu, Z.;Miao, S.;Pan, J.;Shen, L.;Shi, Y.;Li, N. Side-chain structural engineering on poly(terphenyl piperidinium) anion exchange membrane for water electrolysers. *J. Membr. Sci.* **2023**, *665*, 121135.
